# Feminist bioethics in Brazil: development and implications for nursing

**DOI:** 10.1590/0034-7167-2024-0240

**Published:** 2025-06-20

**Authors:** Catarina Flor Silva de Araújo, Sarah de Lima Silva, Graziani Izidoro Ferreira, Dirce Bellezi Guilhem

**Affiliations:** I Universidade de Brasília. Brasília, Distrito Federal, Brazil; II Centro Universitário UNIEURO. Brasília, Distrito Federal, Brazil

**Keywords:** Bioethics, Feminism, Nursing, Brazil, Review., Bioética, Feminismo, Enfermería, Brasil, Revisión.

## Abstract

**Objectives:**

to investigate the implications of the emergence and development of feminist bioethics in Brazil for nursing practice.

**Methods:**

integrative literature review. In April 2023, we searched PubMed, EBSCO, BVS, Web of Science, and Scopus databases, selecting nine studies. Inclusion criteria comprised year and language of publication; we excluded foreign publications and those unrelated to feminist bioethics and nursing.

**Results:**

we identified a gap in research on this topic from 2006 to 2020; most studies discussed the theme of valuing nursing and concluded that political engagement in health is a viable strategy.

**Final Considerations:**

feminist bioethics encourages nurses to discuss gender roles in the workplace and to address vulnerabilities in care delivery. Furthermore, it seeks to promote a more balanced and equitable ethical decision-making process by incorporating the voices of nurses and patients in shaping patient care.

## RESUMEN

**Objetivos:**

investigar las implicancias del surgimiento y desarrollo de la bioética feminista en Brasil para la práctica de enfermería.

**Métodos:**

revisión integrativa de la literatura. En abril de 2023, se buscaron estudios en PubMed, EBSCO, BVS, *Web of Science* y Scopus, eligiendo nueve. Los criterios de inclusión abarcaron año e idioma; se excluyeron estudios extranjeros y sin relación con la bioética feminista y la enfermería.

**Resultados:**

se identificó una brecha en las investigaciones de 2006 a 2020; la mayoría abordó la valorización de la enfermería y concluyó que el compromiso político en salud es una estrategia viable.

**Consideraciones Finales:**

la bioética feminista impulsa a las enfermeras a debatir los roles de género en el trabajo y atender vulnerabilidades en el cuidado. Además, fomenta decisiones éticas más equilibradas e inclusivas al incorporar las voces de enfermeras y pacientes en la construcción del cuidado.

## INTRODUCTION

In response to the rapid advances in medicine and the increasing use of invasive techniques in clinical research from the 1960s to the 1970s, scholars began to emphasize the importance of developing and refining biomedical ethics. This period is marked by the pioneering contributions of Van Rensselaer Potter in his book *Bioethics: A Bridge to the Future* and Henry Beecher’s compilation of critiques on research involving vulnerable human subjects exposed to violence. Early efforts focused on understanding both the issues arising from a lack of standards for medical interventions and bioethics’ potential to transform such situations^(1,2)^.

In 1979, Tom L. Beauchamp and James F. Childress published *Principles of Biomedical Ethics*, a foundational work that solidified and provided tools for bioethics as a field. Building on the work of Potter and Beecher and the guidelines from the Belmont Report for biomedical and behavioral research, their book aimed to create frameworks that protect human dignity^(3)^. Beauchamp and Childress outlined “ethical principles” as moral tools, forming what is still referenced today as the “Principle Theory”. This theory advocated four fundamental pillars for resolving ethical conflicts: autonomy, beneficence, nonmaleficence, and justice. This framework elevated bioethics globally over the next two decades^(2)^.

During the 1990s, various critical bioethical approaches emerged, including feminist-inspired bioethics. This approach, largely developed by female anthropologists, philosophers, and nurses, identified significant flaws within traditional bioethical theories and directed sharp criticism at them^(2)^. For example, they pointed out the persistence of ethical elitism, where decision-making power remained primarily with physicians, sidelining other healthcare team members, patients, and research participants. In opposition, a structured movement advocating ethical plurality and attentiveness to vulnerability gained momentum, termed *Critical Feminist-Inspired Bioethics* ^(4-6)^.

Influenced by second-wave feminism and its focus on defending social minorities, this line of thought introduced the feminist movement’s critical perspective to bioethics, casting a new light on both established issues and marginalized topics within the medical and scientific fields^(5,6)^. Since its inception, feminist bioethics has promoted a critical analysis of ethical principles, emphasizing the need to full apply autonomy and beneficence principles by examining the social and political factors that impact decision-making in health settings^(1)^. This includes advocating for the inclusion of diverse perspectives and organized dissent in ethical debates.

As the feminist bioethics movement evolved, nurses emerged as significant advocates, recognizing the importance of their professional group’s involvement in ethical decision-making, an area previously dominated by medicine. Feminist bioethicists acknowledged for the first time the unique contributions that nurses’ perspectives could provide to the ethics of care in healthcare^(4,5)^. In Brazil, feminist bioethics gained traction in the 1990s, becoming a topic of academic discussion and a component of nursing education^(4)^.

Since feminist bioethics is a relatively new movement gaining increasing prominence in Brazilian academic circles and scientific events in recent years, it is essential to examine the impacts of its consolidation and potential influences on the education and practice of Brazilian nurses. This framework holds relevance not only for bioethics as a discipline but also for the nursing profession. Nurses are increasingly recognized as decision-makers in clinical and research contexts, underscoring their role as a qualified source for bioethical processes.

## OBJECTIVES

To investigate the implications of the emergence and development of feminist bioethics in Brazil for nursing practice.

## METHODS

### Ethical aspects

This research adhered to the guidelines set by Resolution No. 510, dated April 7, 2016, by the National Health Council. As this study exclusively involved scientific texts for the literature review, it did not require registration or evaluation by the Research Ethics Committee (CEP/CONEP)^(7)^.

### Study type

This study is an integrative literature review, a research strategy aimed primarily at promoting Evidence-Based Practice (EBP). This approach involves analyzing literature for results and conclusions related to the chosen research topic and identifying converging points among these studies and their most accurate scientific findings^(8)^.

We employed the Population, Phenomenon of Interest, and Context (PICo) strategy to formulate the following research question: “What are the implications of the emergence and development of feminist bioethics in Brazil for nursing practice, and in what ways do its principles contribute to the process of caregiving in health?”. Here, the corresponding elements are: Population - nursing; Phenomenon of Interest - impacts of feminist bioethics; and Context - caregiving in health.

### Methodological procedures

Inclusion criteria covered articles published in Portuguese, English, or Spanish between 1998 and 2022, considering that the first article on feminist bioethics in a Brazilian journal was published in 1998. We focused on the relationship between feminist bioethics and its connection to nursing. The selected studies were drawn from the following electronic databases: PubMed, EBSCO, Virtual Health Library (VHL), Web of Science, and Scopus. Additionally, some publications were included through manual citation searches in these databases. Articles that did not address nursing, were conducted in other countries, or did not specifically pertain to the feminist branch of bioethics were excluded.

### Data collection and organization

We conducted an online search for scientific material within the aforementioned databases in April 2023, using the following health-related descriptors: feminist bioethics, nursing, nursing care, and Brazil. The search strategy employed Boolean operators as follows: “*Nursing AND feminist ethics AND Brazil; enfermagem AND Bioética feminista AND Brasil; nurse OR nurses OR nursing AND feminist ethics OR feminist bioethics AND Brazil OR Brasil OR Brazilian; Nursing AND feminist ethics AND Brazil*”. We also used the uncontrolled descriptor “feminist bioethics” to account for the historical context of the term and ensure a specific and accurate search.

**Figure 1:**
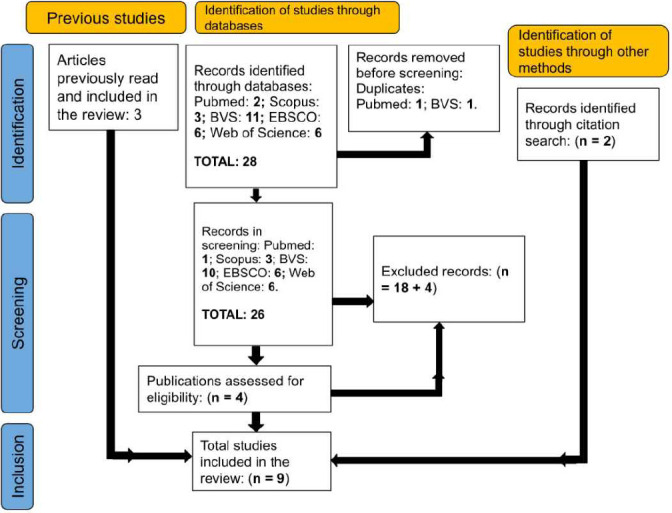
Article selection process for studies included in this review

Source: PRISMA 2020 Flow Diagram^(10)^.

Of the 28 articles identified, we selected those most relevant to the study that met the previously established inclusion criteria. We utilized the online tool Rayyan^(9)^ to remove two duplicate articles. Titles and abstracts of the studies were then reviewed, leading to the exclusion of an additional 18 publications that did not align with the study’s objectives and theme. The remaining eight articles were subjected to full-text review and further selection, with four chosen for analysis. We added three previously reviewed articles relevant to the study and two more that were found through citation searches. In total, nine articles were included for analysis (Figure 1). We used Office 360 online software to organize and document the reading and selection process.

### Data analysis

The nine selected articles underwent critical content analysis according to the method outlined by Cardoso et al., 2021^(11)^. The findings and conclusions of these publications were compared and evaluated to identify points of convergence and divergence, thereby establishing evidence that addresses the research question.

The data collection focused on the following categories: publication year; study design; main issues presented; and conclusions and potential solutions.

## RESULTS

We categorized the texts by critically comparing each study’s findings through discussions among the authors (Chart 1).

**Chart 1:** Studies included in the sample, detailing title, authors, year; design; main issues; and conclusions and potential solutions

Title; authors; year	Design	Main issues	Conclusions and potential solutions
“*Bioética Feminista: a emergência da diferença*”; Diniz, Vélez; 1998^(12)^	Narrative review	Nurses are seen as the most suitable figures for decision-making in end-of-life care, as they establish close bonds with patients during the caregiving process, thus gaining a profound understanding of them. However, they are often subordinated, submissive, and passive to the male figure of the physician, who holds objective moral authority.	Through feminist bioethics, it is essential to challenge the universalizing and neoliberal nature of traditional bioethics, disregarding vulnerable groups and supporting individualism. The goal is to amplify marginalized and vulnerable voices.
“*Bioética Feminista: O Resgate Político do Conceito de Vulnerabilidade*”; Diniz, Guilhem; 1999^(13)^	Narrative review	Feminist bioethics critiques the “marriage” of bioethics and medicine, where the discussion is restricted to medical interests, limiting inquiries and silencing other voices, such as the nurse’s. The ethical principle of autonomy is questioned when it is subverted to control female bodies.	Allowing a plurality of perspectives does not imply accepting radical relativism. Ethical boundaries must still be established to ensure that new inequities are not tolerated. For feminist bioethics, the involvement of nurses in mediating these discussions is essential and should work to dismantle conventional power structures that exclude this group based on race, class, and gender discrimination.
“*Mulheres e Profissionais de Saúde: O Imaginário Cultural na Humanização ao Parto e Nascimento*”; Griboski, Guilhem; 2006^(14)^	Qualitative research with content analysis, n = 45	Significant gaps exist in the knowledge of birthing women about the childbirth process, the physiological functioning of their own bodies, and reproductive rights. Additionally, nurses’ subordination in the hospital context is highlighted as an important factor perpetuating gender roles that delegate decision-making power to men.	Change should begin within universities through the conscious training of healthcare professionals, particularly nurses, who are aligned with values historically associated with the profession and femininity (such as care, empathy, and compassion), especially in support of women who use health services for childbirth.
“Are Submissive Nurses Ethical? Reflecting on Power Anorexia”; Lunardi, Peter, Gastaldo; 2002^(15)^	Narrative review	Nurses lack political awareness; many do not recognize or believe in the political significance of their daily work. Foundational factors (class, ethnicity, and gender) define the boundaries of freedom of choice, making a generalized approach to autonomy inherently unjust.	Concrete changes are needed in workplace environments to give nurses sufficient space to exercise their authority. However, this ideal can only be achieved by politicizing nurses, enabling them to recognize their collective power and unite as a group to advocate for their interests.
“The practice of nurses in the implementation of public policies for the black population: in the light of feminist ethics”; Rezende, et al; 2021^(16)^	Single, integrated case study of a qualitative nature	The intersectionality of minority groups shapes power dynamics. The movement toward identity recognition and cultural appreciation is also integral to implementing care grounded in this ethical perspective.	Nursing work holds political and social empowerment potential by enabling patients to become active agents in their own healthcare and fostering awareness of their rights. Building an honest and shared knowledge relationship is crucial to achieving this empowerment.
“Gender and Empowerment by Nursing Students: Representations, Discourses and Perspectives”; Nogueira, Spagnol, Rocha, et al; 2022^(17)^	Exploratory qualitative research, n = 12	The marginalization of nursing due to the sexual division of labor reinforces the biomedical health model. There is an attempt to subordinate nursing work to that of medicine. Moreover, Black women experience an even greater lack of recognition, including from white colleagues within the profession.	The transformation should start in universities. Discussions about gender consciousness and roles within academic training must result in professional recognition, the establishment of new roles, and improved conditions, considering intersecting factors such as race and class.
“Ethics, COVID-19 and nursing vulnerability: analysis of photographs released by the media”; Sena, Fontenele, Duarte, et al; 2022^(18)^	Documentary study with a qualitative approach, n = 74	Brazilian nursing is predominantly made up of Black and mixed-race women who face daily silencing and violence amid precarious working conditions, denial of rights, and discrimination. These vulnerabilities were further exacerbated during the current humanitarian crisis.	Increasing the profession’s visibility in society is necessary to gain recognition as competent, ethically sound, and scientifically grounded. Professional appreciation should also include ensuring improved working conditions.
“*A redescoberta da ética do cuidado: o foco e a ênfase nas relações*”; Zoboli; 2004^(19)^	Narrative review	There is a devaluation of the feminine ethics of nursing care, which emphasizes non-violent conflict resolution and collective notions of care and responsibility.	Promoting feminist ethics facilitates the pursuit of diverse perspectives by incorporating historically excluded and oppressed voices, aiming to enable political and social changes.
“*Aborto, Objeção de Consciência e Bioética Feminista: Estratégias para Efetivação do Direito à Interrupção Legal da Gestação*”; Moreira, Oliveira; 2020^(20)^	Bibliographic and documentary study	Nursing plays a crucial role in guiding women through the process of legal abortion, from entry to discharge. It is common for these professionals to exercise conscientious objection only when the health team is fully staffed, ensuring that their choice does not compromise patient care.	The authors highlight the importance of mechanisms that protect both the right to conscientious objection and the patient’s right to care, ensuring that patients are not adversely affected in this process. Protecting women’s rights should guide these decisions.

### Publication period

The articles analyzed were published between 1998 and 2022. Four of these studies were published between 2020 and 2022, while the remaining five appeared between 1998 and 2006, indicating a significant hiatus on the topic in Brazil between 2006 and 2020.

### Study design

Four narrative reviews, two qualitative studies, two documentary studies, and one case study were among the studies analyzed.

### Main issues

Regarding the topics covered, six articles addressed “professional recognition of nursing care”; five articles approached “power and gender relations in nursing”; four publications discussed “intersectionality of class, race, and gender”; and two articles discussed “human reproduction” and “abortion”, either individually or jointly.

### Conclusions and potential solutions

Among the proposed solutions, the topic of “politicization in health” was prominent, appearing in seven articles. All of these identified “awareness-building in universities” as a focal point. The remaining two articles highlighted “attention to vulnerabilities” as a key conclusion.

## DISCUSSION

In Brazil, feminist-inspired bioethics has mirrored the international movement to broaden the scope of debates on morally complex issues. This approach emphasizes pluralism, gender issues, social and professional class distinctions, and the inclusion of individuals traditionally marginalized or excluded from ethical discussions-such as women, the economically disadvantaged, children, and older people^(6,21-23)^. Since the 1990s, recurring themes within feminist bioethics have included: 1) reproductive issues, such as sexual and reproductive rights, including assisted reproduction; 2) ethics in public health, focusing on access to services and health inequities on national and global levels; 3) disability and personhood, more recently through the lens of human rights and the social model of disability; 4) psychiatry and mental health, especially examining mental healthcare for women; 5) inclusion of women in clinical research; and 6) autonomy and shared decision-making, among other pertinent topics^(21,24)^.

Feminist bioethics has emerged as an intersectional and intersectoral approach, aiming to address the challenges posed by diversity, differences, and varying levels of vulnerability inherent in society^(5,25)^. In nursing, these themes are reflected in daily practices related to patient care, interpersonal and interprofessional relationships, empowerment of nursing professionals^(2,18)^, and advocacy for the rights of patients and families, providing support for shared decision-making processes^(26)^. Thus, autonomy emerges as a crucial principle, empowering both patients and the nursing staff within the healthcare team.

Since the earliest studies on feminist bioethics in Brazil, nursing has played a prominent role, both in producing research on the subject and in practically applying its concepts and ideals^(5,6)^. This feminist and critical bioethical perspective seeks to challenge the status quo of medical paternalism and amplify the voices of those historically silenced in clinical and hospital contexts^(12,13)^. Consequently, nurses are recognized for their transformative potential, closely associated with patient care. Therefore, this professional group is uniquely capable of absorbing, analyzing, and recalibrating biomedical logic based on each individual’s real needs and contexts, a perspective supported by numerous articles reviewed^(14-18)^.

As a historically female profession with a consistent struggle for rights, nursing faces daily ethical and moral issues that reflect broader gender and power debates in public and political spheres^(13,17)^. Given the inherently transformative nature of these issues within professional practice, it is essential to examine workplace dynamics influenced by gender roles, the undervaluation of caregiving, the impact of social intersectionality on care processes, and nursing’s pivotal role in supporting human processes, including birth and end-of-life care^(13-19)^.

Continuous, patient-centered care is intrinsic to nursing, and nurses play a vital technical role in organizing healthcare services, including the logistical distribution of materials, managing care needs, and prioritizing urgent cases^(15,19)^. Nurses are acknowledged as responsible for direct care and maintaining essential elements for the effective functioning of the entire healthcare team^(15,19)^. However, paradoxically, this group is often denied recognition for its moral responsibility and potential to contribute to ethical decision-making-a role typically reserved for physicians. The analysis of this dynamic through the lens of feminist bioethics reveals the marginalization of the nursing profession and the consequent impoverishment of the ethical debate due to due to this group’s exclusion^(12,13,15,18)^.

The historical subjugation of nurses and their relegation to a position of passivity and submission to the male figure of the physician-who is considered the moral authority-creates the illusion that nursing is subordinate to medical practice^(12,19)^. This context highlights the power dynamics related to gender issues in healthcare, where marginalizing nursing judgment, or the ethics of care^(19,27)^, effectively removes the nurse-the female figure-from ethical discourse^(12,13)^.

The subjugation of feminine morality originated and was widely propagated in the 1970s with early studies on moral development and ethical thinking^(27)^. This perspective developed partly because early researchers and study participants were exclusively male, leading to the assumption that men were morally mature and thus suitable for ethical decision-making. Women, conversely, were deemed morally immature and expected to conform. In contrast to these views, men and women may approach ethical decisions differently, with men associated with the ethics of justice and women with the ethics of care. The juxtaposition of these two models underscores the necessity of reevaluating dominant behavioral and decision-making standards and giving voice to those historically silenced. This recognition fosters the moral pluralism essential to contemporary society^(27)^.

Understanding the historical connection between the feminine figure and the nurse’s role-and how this association extends into the healthcare work environment-is crucial. Traditionally, clinical and ethical debates have been restricted to those perceived as moral authorities, who are thus deemed capable of sound decision-making, reflecting socially constructed gender roles in these settings. Associating nursing work with inherent kindness and a natural vocation has had severe implications for the development of this system. Nurses were expected to embody obedience and compassion but not to exercise decision-making or leadership^(28,29)^.

A significant issue highlighted within these relationships is the impact of intersecting identities related to race, class, and gender, which leads to even greater neglect and silencing of Black women-whether they are healthcare users or even members of the nursing team^(17,18)^. From an intersectional perspective, this construct can essentially be defined as examining how power dynamics shaped by gender, ethnicity, and class, among others, influence both the socially diverse public sphere and individuals’ daily personal lives^(30)^. Within this framework, various forms of discrimination and privilege are understood to operate not in isolation but in overlapping ways that influence individuals’ holistic experiences.

Institutional racism experienced by Black and brown women manifests, for example, in their limited access to healthcare services, permeating the entire care process, underscored by a silent acknowledgment of inequities^(31)^. Within institutions reflecting racial and gender dynamics, the healthcare needs of these women are often ignored. This situation has severe consequences, as demonstrated in the “*Nascer no Brasil* II: National Survey on Abortion, Birth, and Delivery”^(32)^, which reported that, between 2022 and 2023, more Black women than white women died postpartum due to preventable factors, such as late prenatal care and pregnancy-related conditions.

The power dynamics stemming from intersecting identities among minority groups determine who is empowered to speak and whose voices are heard^(16)^. Even within the nursing profession, Black and Indigenous nurses who have contributed immensely to the field often face historical and documented marginalization. An example is Mary Jane Seacole, a Jamaican nurse contemporary to Florence Nightingale, who is rarely acknowledged or cited in introductory nursing courses worldwide^(33)^.

Furthermore, during recent events like the COVID-19 pandemic, there were numerous attempts to erase and silence the contributions of Black and brown female nurses and nursing technicians. Media and social media platforms frequently highlighted white male figures, underrepresenting the predominance of female and nonwhite professionals in the healthcare workforce^(18)^. Women, by contrast, are assigned positions with less social prestige, lower pay, and minimal decision-making power-roles where the identities of “good professional” and “good woman/wife/mother” are expected to intersect^(34)^.

During health crises such as the COVID-19 pandemic^(28)^ and Ebola epidemics^(35)^, women are routinely called upon to sacrifice themselves in the name of caring for others, including the community, children, and men. For women, the fear of contamination and illness during such crises is compounded by increased household and childcare responsibilities due to school and daycare closures, heightened family demands for emotional presence, and increased exposure to domestic violence-factors that contribute to physical and psychological distress. For healthcare professionals, expanded domestic caregiving responsibilities are compounded by professional care responsibilities for the community, which often involve the same expectations of emotional support and attentiveness. Despite the stark mental and physical workload endured by these professionals, they continue to face inadequate recognition, pay, and moral silencing within the profession^(36)^.

Understanding this dynamic invites critical examination of why nursing-despite being the majority in healthcare and responsible for managing patient care processes-continues to struggle for recognition and faces frequent attempts at professional subordination^(12,13,15,17,19,20)^. As Nogueira et al.^(17)^ highlight, even after years of collective feminist advocacy, nurses encounter substantial barriers to achieving leadership positions-not only within the broader healthcare field but also within their own decision-making bodies, where political representation is still male-dominated. Women comprise roughly 70% of the healthcare workforce globally yet occupy only about 25% of leadership roles^(29)^.

This phenomenon, referred to as the *power anorexia in nursing* ^(15)^, has also been linked to a diminished sense of autonomy among patients, who are similarly subordinated to medical decisions and often have their voices silenced^(14,20)^. In contexts such as childbirth, abortion, and euthanasia^(12-14,20)^, this power imbalance is evident, even in these sensitive situations. Patient autonomy is undermined when it conflicts with the needs and beliefs of the medical team, who often hold the “final say” in ethical deliberations. In other words, the social and psychological aspects that influence the application of patient autonomy-well understood by feminist bioethicists^(12,13)^-are disregarded, leading to the exclusion of nursing professionals who could interpret and respond to these patient needs during care.

Moreover, a culture of compulsory female submission, in which violence and pain are embedded in hospital processes, persists, perpetuated by a depoliticized healthcare system and reinforced by a technicist, paternalistic biomedical model^(14)^. Only by acknowledging the role of gender, social, and class dynamics-intersectionalities that shape patients’ choices and needs, as well as health education-can we work toward the full realization of patient autonomy^(15-17)^. As one article aptly noted, autonomy cannot exist without knowledge^(13)^.

It is crucial to scrutinize the application of the autonomy principle, particularly among women who undergo invasive reproductive techniques, to prevent coercion from being masked by rhetoric that ultimately enables violence^(13)^. Evaluating not only the choices made but also how those choices were reached is essential^(12,13)^. In settings where clinical paternalism is predominant and patients are inadequately informed about their health conditions, treatment options, and potential outcomes, it is evident that decision-making occurs solely among professionals, with patients’ personal experiences, feelings, and opinions left unheard^(37)^.

Studies have found^(14-20)^ that ethical principles should not be applied universally without considering individual contexts and shared privileges. Some articles demonstrated that initiating these discussions within nursing schools is essential for driving systemic changes^(14,17)^. It is urgent to promote discourse and politicization, examining the intersections of gender, race, and class^(17)^, but without limiting this dialogue to academic institutions; structural and policy changes are needed to solidify nursing’s power in practice^(15,18)^.

Additionally, these studies underscore that the work of raising social and political awareness should extend to support vulnerable groups, with nurses playing a vital role in empowering individuals within the healthcare system to become active agents of their own health and advocates for their rights^(14-16)^. One study^(16)^ reported a successful example of integrating cultural elements from the community into nursing care, which improved public engagement with the service and fostered an environment for learning and awareness. Building honest, trust-based relationships in care is essential to achieving these outcomes^(14,16)^.

### Study limitations

One limitation of this study was the constraint of a fixed period for searching articles within open-access databases or databases accessible through the educational institution. Additionally, finding articles addressing both nursing practice and feminist bioethics was challenging. Furthermore, the significant scarcity of published articles on the subject, even with the use of the uncontrolled descriptor “feminist bioethics”, limited the analysis to a small number of articles. This scarcity represents a critical gap in current knowledge, which hindered the depth and discussion of the results.

### Contributions to the field of nursing

This study seeks to enrich the bioethical debate by encouraging the questioning and reconsideration of nurses’ and patients’ voices being excluded from the discussion-especially those in vulnerable situations. We hope the ideas discussed here will lead to the development and implementation of initiatives that result in practical changes in care, enhancing working conditions for women in healthcare and optimizing care with equity and respect for different perspectives, experiences, and expressions of health.

## FINAL CONSIDERATIONS

Nursing remains impacted by critical issues related to gender, the sexual division of labor, and the political marginalization of women in the profession. The ambivalent dynamic, where nurses are simultaneously made vulnerable within healthcare teams and hold significant transformative potential for the patients they serve, highlights the complexity of this discussion. More importantly, it underscores the importance of politicizing the group in the fight for greater representation and advocacy for the interests and needs of vulnerable groups.

Recognition of intersectionalities and respect for cultural diversity in care help mitigate the effects of gender, class, and racial inequalities. From this perspective, valuing the collective knowledge built through broad dialogue enriches the ethical debate and reaches those previously silenced. Therefore, strengthening the nursing profession also means strengthening the community.

In conclusion, feminist bioethics does not advocate that nurses assume the paternalistic role traditionally held by the medical establishment and make decisions for those they care for. Instead, an equitable and balanced ethical deliberation process should ensure that both nurses and patients are heard, considered in their diverse contexts, and regarded as active participants in care development.
