# Supplementary material for: Feminist bioethics in Brazil: development and implications for nursing
Source: Rev Bras Enferm. 2025 Jun 20;78(2):e20240240. doi: 10.1590/0034-7167-2024-0240 (PMC12185139; doi:10.1590/0034-7167-2024-0240)
Supplement: Translation

# Translation: Bioética feminista no Brasil: desenvolvimento e implicações para a Enfermagem

**DOI:** 10.1590/0034-7167-2024-0240pt

**Authors:** Catarina Flor Silva de Araújo, Sarah de Lima Silva, Graziani Izidoro Ferreira, Dirce Bellezi Guilhem

**Affiliations:** I Universidade de Brasília. Brasília, Distrito Federal, Brasil; II Centro Universitário UNIEURO. Brasília, Distrito Federal, Brasil

**Keywords:** Bioética, Feminismo, Enfermagem, Brasil, Revisão.

## Abstract

**Objetivos:**

investigar as implicações do surgimento e desenvolvimento da bioética feminista no Brasil para a prática de enfermagem.

**Métodos:**

revisão integrativa da literatura. Utilizaram-se as bases de dados PubMed, EBSCO, BVS, *Web of Science* e Scopus em abril de 2023, sendo eleitos nove estudos. Os critérios de inclusão abarcaram ano e língua de publicação; excluíram-se publicações estrangeiras e as não pertinentes à bioética feminista e enfermagem.

**Resultados:**

identificou-se lacuna de produções na temática entre 2006 e 2020; a maioria dos estudos discutiu o tópico de valorização da enfermagem e concluiu como uma solução a estratégia de politização em saúde.

**Considerações Finais:**

a bioética feminista propõe entre as enfermeiras a discussão acerca dos papéis de gênero no trabalho e atenção às vulnerabilidades na assistência. Além disso, busca promover um processo mais equilibrado e equitativo nas deliberações éticas mediante a escuta e inserção de enfermeiras e pacientes na construção do cuidado.

## INTRODUÇÃO

Diante dos rápidos avanços da medicina e do uso de técnicas cada vez mais invasivas na pesquisa clínica entre as décadas de 1960 e 1970, estudiosos buscaram evidenciar a importância de construir e refinar uma ética biomédica. O destaque vai para o pioneirismo das produções de Van Renssealaer Potter no livro “Bioética: Uma ponte para o futuro” e de Henry Beecher e seu compilado de denúncias sobre pesquisas envolvendo seres humanos em situação de vulnerabilidade e violência. Nesse primeiro momento, buscava-se compreender tanto os problemas resultantes da falta de métricas para aplicar intervenções médicas quanto o potencial da bioética na transformação desses cenários^(1,2)^.

Em 1979, Tom L. Beauchamp e James F. Childress publicaram o livro “Princípios da Ética Biomédica”, marco para a consolidação e instrumentalização da Bioética. Eles basearam-se nas propostas de Potter e Beecher bem como nas diretrizes indicadas no Relatório Belmont para pesquisas biomédicas e comportamentais, que buscava a construção de ferramentas de preservação da dignidade humana^(3)^. Beauchamp e Childress apontaram “princípios éticos” que deveriam ser utilizados como ferramentas morais e referenciados ainda hoje. Conhecida como “Teoria dos Princípios”, a tese dos autores defendia a importância de quatro pilares básicos para a resolução de conflitos éticos: autonomia, beneficência, não maleficência e justiça. Essa primeira teoria alavancou a Bioética ao redor do mundo pelas duas décadas posteriores^(2)^.

Nos anos 1990, emergiram as bioéticas críticas, dentre as quais a Bioética de Inspiração Feminista. Esta foi uma linha sobre a qual se debruçaram principalmente antropólogas, filósofas e enfermeiras^(2)^. Apontaram problemas importantes na tese e passaram a lhe dirigir duras críticas, como a manutenção do elitismo ético em que o poder de decisão permanece nas mãos dos médicos, excluindo outros membros da equipe de saúde, pacientes e participantes de pesquisa. Opondo-se a isso, cresceu um movimento estruturado de ideias pautadas na pluralidade ética e na atenção às vulnerabilidades, denominado “Bioética Crítica de Inspiração Feminista”^(4-6)^.

Influenciada pela segunda onda do feminismo e sua preocupação com a defesa de minorias sociais, essa corrente de pensamento trouxe para o campo bioético a criticidade própria do movimento feminista como uma lupa sobre as questões já discutidas e sobre temáticas ainda marginalizadas pelo corpo médico e científico^(5,6)^. Desde sua criação, a bioética feminista tem proposto a análise crítica dos princípios éticos, ressaltando a necessidade da discussão acerca da aplicação plena dos princípios da autonomia e da beneficência, por meio da investigação dos fatores sociais e políticos que influenciam as decisões no campo da saúde^(1)^. Nesse sentido, a defesa da inclusão de novos discursos e diferentes vozes no debate e da dissonância organizada são temas muito bem-vindos.

No processo de construção e difusão do movimento, destacou-se o protagonismo de enfermeiras que compreendiam a importância de seu grupo ser incluído na tomada de decisão ética, até então dominada pela medicina. Entre as bioeticistas, pela primeira vez reconheceu-se o potencial inovador que as vozes provenientes dessa categoria profissional poderiam agregar conhecimento sobre a eticidade do cuidar em saúde^(4,5)^. No Brasil, a partir da década de 1990, a temática ganhou força e passou a compor o rol de teorias debatidas na academia e no ensino de enfermagem^(4)^.

Tratando-se de um movimento relativamente novo e que tem ganhado crescente notoriedade nos meios acadêmicos e em eventos de propagação científica no Brasil nos últimos anos, torna-se importante analisar os efeitos de sua consolidação e possíveis influências para a formação e prática de enfermeiras brasileiras. Essa corrente de pensamento mostra-se relevante não apenas para a bioética como disciplina, mas também para a classe da enfermagem. Tal categoria assume prerrogativa de tomada de decisão nos cenários de práticas clínicas e de pesquisa, o que demonstra o reconhecimento dessas profissionais como fonte qualificada para os processos de caráter bioético.

## OBJETIVOS

Investigar as implicações do surgimento e desenvolvimento da bioética feminista no Brasil para a prática de enfermagem.

## MÉTODOS

### Aspectos éticos

A presente pesquisa cumpriu as normas estabelecidas pela Resolução Nº 510, de 07 de abril de 2016, do Conselho Nacional de Saúde. Foi realizada exclusivamente com textos científicos que compuseram a revisão da literatura, dispensando o registro e a avaliação pelo sistema CEP/CONEP^(7)^.

### Tipo de estudo

Trata-se de uma revisão integrativa da literatura. Essa estratégia de pesquisa objetiva principalmente a promoção da Prática Baseada em Evidências (PBE), em que se busca, na literatura, os resultados e conclusões produzidas sobre o tema de pesquisa escolhido e estabelece os pontos de convergência entre esses estudos e suas informações científicas mais acuradas^(8)^.

Neste estudo, utilizou-se a estratégia População, Fenômeno de Interesse e Contexto (PICo) para nortear a construção da seguinte pergunta de pesquisa: “Quais são as implicações do surgimento e desenvolvimento da bioética feminista no Brasil para a prática de enfermagem e de que maneira seus princípios contribuem para o processo de cuidar em saúde?”. Dessa forma, as correspondências são: População - enfermagem; Fenômeno de Interesse - impactos da bioética feminista; e Contexto - processo de cuidar em saúde.

### Procedimentos metodológicos

Os critérios de inclusão estabelecidos abrangeram artigos publicados em português, inglês ou espanhol, entre 1998 e 2022, considerando a data de publicação (1998) do primeiro artigo sobre bioética feminista em um periódico brasileiro. Observou-se a relação entre bioética feminista e seu vínculo com a enfermagem. Consideraram-se as publicações existentes nas seguintes de bases de dados eletrônicas: PubMed, EBSCO, Biblioteca Virtual em Saúde (BVS), *Web of Science* e Scopus. Além disso, algumas publicações foram incluídas por meio de busca manual pela citação nas bases de dados. Foram excluídos os artigos que não abordavam a enfermagem, aqueles realizados em outros países e os que não tratavam especificamente da corrente feminista da bioética.

### Coleta e organização dos dados

Realizou-se a busca on-line do material científico nas bases de dados supracitadas durante o mês de abril de 2023, empregando-se os seguintes descritores em saúde: bioética feminista, enfermagem, cuidado de enfermagem e Brasil. Utilizou-se a seguinte estratégia de pesquisa com auxílio de operadores booleanos: “*Nursing AND feminist ethics AND Brazil; enfermagem AND Bioética feminista AND Brasil; nurse OR nurses OR nursing AND feminist ethics OR feminist bioethics AND brazil OR brasil OR brazilian; Nursing AND feminist ethics AND Brazil*”. Foi usado o descritor não controlado “bioética feminista”, considerando a historicidade do termo e a necessidade de tornar a busca a mais específica e acurada possível.

Dentre os 28 artigos encontrados, foram selecionados aqueles de maior relevância para o estudo e que atendiam aos critérios de inclusão previamente definidos. Utilizou-se a ferramenta on line Rayyan^(9)^, excluindo-se dois artigos duplicados. Foi efetuada a leitura de títulos e resumos dos estudos, tendo sido excluídos mais 18 publicações que não se enquadravam nos objetivos e tema do estudo. Finalmente, os oito artigos restantes foram submetidos à leitura na íntegra e nova seleção, sendo quatro escolhidos para compor a análise. Somados a estes, foram adicionados ainda três artigos anteriormente lidos e incluídos na pesquisa e dois outros encontrados e selecionados por meio de busca por citação. Ao todo, foram nove artigos para análise (Figura 1). Foram usados programas on line do Pacote Office 360 para organização e registro do processo de leitura e seleção de estudos.

**Figura 1:** Processo de seleção dos artigos incluídos no estudo

*Fonte: Fluxograma Prisma 2020^(10)^.*

### Análise dos dados

Os nove artigos selecionados foram submetidos à análise crítica de conteúdo, conforme Cardoso et al., 2021^(11)^. Os resultados e conclusões dessas publicações foram comparados e avaliados para estabelecer pontos de convergência e divergência e estabelecer as evidências que respondessem à pergunta de pesquisa.

Para coleta de informações, observaram-se as seguintes categorias: ano de publicação; delineamento do estudo; principais problemáticas apresentadas; e conclusões e possíveis soluções.

## RESULTADOS

Os textos foram categorizados com base na comparação crítica dos achados de cada estudo por meio do debate entre os autores (Quadro 1).

**Quadro 1:** Estudos incluídos na amostra com informações sobre título, autores e ano; delineamento; problemáticas principais; e conclusões e possíveis soluções

Título; autores; ano	Delineamento	Problemáticas principais	Conclusões e possíveis soluções
“Bioética Feminista: a emergência da diferença”; Diniz, Vélez; 1998^(12)^	Revisão narrativa	A enfermeira é lida como a figura mais adequada para a tomada de decisão no processo de morte, uma vez que essa profissional estabelece vínculo no decorrer do cuidado, conhecendo profundamente os pacientes. São subjugadas, submissas e passivas à figura masculina do médico, que possui a moral objetiva.	Por meio da bioética feminista, deve haver o questionamento do caráter universalizante e neoliberal da bioética tradicional, que desconsidera os grupos vulneráveis e apoia o individualismo. O objetivo é trazer à luz as vozes marginalizadas e vulneráveis.
“Bioética Feminista: O Resgate Político do Conceito de Vulnerabilidade”; Diniz, Guilhem; 1999^(13)^	Revisão narrativa	A bioética feminista denuncia o “casamento” bioética-medicina, em que o debate se restringe aos interesses médicos, o que limita os questionamentos e impede que outras vozes se manifestem, como a da enfermeira. Questiona-se o cumprimento da autonomia quando há a subversão desse princípio ético para controle de corpos femininos.	Possibilitar a pluralidade dos discursos não significa aceitar o relativismo radical. É preciso ainda que limites éticos sejam estabelecidos a fim de que novas iniquidades não sejam toleradas. Para a bioética feminista, a participação das enfermeiras nessa mediação é fundamental e deve ser exercida de maneira a desconstruir as estruturas de poder convencional que excluem essa classe por meio das discriminações de raça, classe e gênero.
“Mulheres e Profissionais de Saúde: O Imaginário Cultural na Humanização ao Parto e Nascimento”; Griboski, Guilhem; 2006^(14)^	Pesquisa qualitativa com análise de conteúdo, n = 45	Há lacunas no conhecimento das mulheres em situação de parto sobre a parturição, funcionamento fisiológico de seu próprio corpo e seus direitos reprodutivos. Além disso, a subordinação e submissão das enfermeiras no contexto hospitalar é questionada e apontada como fonte importante de reprodução de papéis de gênero que delegam o poder de escolha para o masculino.	A mudança deve começar nas universidades, na formação consciente dos profissionais de saúde, sobretudo de enfermeiras, identificadas com os valores historicamente associados à classe e ao feminino (p.ex., cuidado, empatia e compaixão) a favor das mulheres que utilizam o serviço de saúde para parir.
“*Are Submissive Nurses Ethical? Reflecting on Power Anorexia*”; Lunardi, Peter, Gastaldo; 2002^(15)^	Revisão narrativa	Há a despolitização das enfermeiras ao passo em que estas não reconhecem seu poder no exercício diário de seu trabalho, ou não acreditam que seu trabalho também é político. Fatores de base (classe, etnia e gênero) ditam as fronteiras da liberdade de escolha, portanto o processo de autonomia generalizado é injusto.	É necessária a mudança concreta dos ambientes de trabalho, de forma que enfermeiras tenham espaço suficiente para exercer seu poder. Porém, esse ideal só será conquistado mediante a politização das enfermeiras, reconhecendo-se no coletivo e unindo-se enquanto grupo para lutar por seus interesses.
“*The practice of nurses in the implementation of public policies for the black population: in the light of feminist ethics*”; Rezende, et al; 2021^(16)^	Estudo de caso único e integrado de natureza qualitativa	A interseccionalidade de grupos minoritários constrói as relações de poder. O movimento de identificação e de valorização cultural também faz parte da implementação do cuidado pautado nessa ética.	O trabalho de enfermagem tem potencial de empoderamento político e social, ao tornar os pacientes agentes de sua própria saúde e estimular a consciência sobre seus direitos. Para isso, é necessária a construção de uma relação honesta e compartilhada de conhecimentos.
“*Gender and Empowerment by Nursing Students: Representations, Discourses and Perspectives*”; Nogueira, Spagnol, Rocha, et al; 2022^(17)^	Pesquisa qualitativa exploratória, n = 12	A marginalização da enfermagem pela divisão sexual do trabalho perpetua o modelo biomédico de saúde. Existe a tentativa de subordinação do trabalho de enfermagem ao da medicina. Além disso, para mulheres pretas, a falta de reconhecimento é ainda maior, mesmo entre as colegas de profissão brancas.	A mudança deve começar nas universidades. A valorização da profissão, a conquista de novos espaços e melhora das condições devem vir do debate a respeito da construção da consciência de gênero e dos papéis de gênero presentes na formação acadêmica, que atravessam também fatores como raça e classe.
“*Ethics, COVID-19 and nursing vulnerability: analysis of photographs released by the media*”; Sena, Fontenele, Duarte, et al; 2022^(18)^	Estudo documental, com abordagem qualitativa, n = 74	A enfermagem brasileira é composta majoritariamente por mulheres pretas e pardas, que enfrentam diariamente o silenciamento e a violência em meio a condições de trabalho precárias, negação de direitos e discriminação. Durante a maior crise humanitária da atualidade, essas vulnerabilidades exacerbaram-se.	É preciso aumentar a visibilidade da classe perante a sociedade, para que possa ser reconhecida profissionalmente como competente e dotada de princípios éticos e científicos. A valorização deve passar também pela garantia de melhores condições de trabalho.
“A redescoberta da ética do cuidado: o foco e a ênfase nas relações”; Zoboli; 2004^(19)^	Revisão narrativa	Observa-se a desvalorização da ética feminina do cuidado de enfermagem, que se centra na resolução não violenta de conflitos e na importância à noção de cuidado e responsabilidade coletivos.	O fomento da ética feminista permite a busca pela pluralidade dos discursos com a introdução das vozes historicamente excluídas e oprimidas, visando viabilizar mudanças político-sociais.
“Aborto, Objeção de Consciência e Bioética Feminista: Estratégias para Efetivação do Direito à Interrupção Legal da Gestação”; Moreira, Oliveira; 2020^(20)^	Estudo bibliográfico e documental	A enfermagem tem papel relevante como porta de entrada, acompanhamento e saída das mulheres no serviço de abortamento legal. Comumente, observa-se que essas profissionais se preocupam em apenas utilizar‑se da objeção de consciência quando a equipe de saúde está completa, garantindo que sua escolha não afete o atendimento da paciente.	Os autores apontam a importância de mecanismos que garantam o direito à objeção concomitantemente ao direito ao atendimento da usuária do serviço, que de forma alguma deve ser prejudicada neste processo. A proteção dos direitos da mulher deve guiar as decisões.

### Período das publicações

Foram encontrados artigos produzidos entre 1998 e 2022, sendo que quatro foram publicados entre 2020 e 2022, e o restante, cinco estudos, de 1998 a 2006, evidenciando um período de hiato importante sobre a temática no Brasil entre 2006 e 2020.

### Delineamento dos estudos

Dentre os estudos analisados, quatro eram revisões narrativas, dois eram pesquisas qualitativas, dois eram estudos documentais, e um era estudo de caso.

### Problemáticas principais

Quanto aos temas abordados nos estudos, “valorização profissional do cuidado de enfermagem” está presente em seis artigos; “relações de poder e gênero na Enfermagem” aparece em cinco artigos; “interseccionalidades de classe, raça e gênero” é discutido em quatro publicações; e “reprodução humana” e “aborto” são discutidos em dois artigos, sendo que os temas podem aparecer conjugados ou não nas publicações.

### Conclusões e possíveis soluções

Entre as possíveis soluções apresentadas nos estudos, o tópico “politização em saúde” foi recorrente, aparecendo em sete artigos; dentre estes, todos apontaram a “conscientização nas universidades” como ponto focal. Os demais artigos (n = 2) abordaram a “atenção às vulnerabilidades” como conclusão importante.

## DISCUSSÃO

No Brasil, a Bioética de Inspiração Feminista tem acompanhado o movimento internacional para introduzir e aproximar temas que ampliem o cenário para o debate de questões de difícil mediação moral. A introdução do pluralismo, questões de gênero, classes sociais e profissionais e visibilidade às deficiências e a pessoas tradicionalmente oprimidas e excluídas dos debates éticos (p.ex., mulheres, pobres, crianças, idosos, entre outros) são assuntos muito caros nesse contexto^(6,21-23)^. Temas recorrentes desde o seu surgimento na década de 1990 estão relacionados a: 1) questões reprodutivas: direitos sexuais e reprodutivos, incluindo reprodução assistida; 2) ética em saúde pública: acesso aos serviços e iniquidades em saúde nacional e globalmente; 3) deficiência e personificação: modelo social da deficiência, mais recentemente sob a ótica dos direitos humanos; 4) psiquiatria e saúde mental: exame crítico do atendimento em saúde mental, especialmente para as mulheres; 5) inclusão de mulheres em pesquisas clínicas; e 6) autonomia e decisões compartilhadas, entre outros temas que poderiam ser mencionados^(21,24)^.

É possível verificar que a Bioética Feminista emergiu de forma interseccional e intersetorial buscando vencer desafios impostos pela diversidade, diferenças e distintos níveis de vulnerabilidade presentes na vida em sociedade^(5,25)^. Na sua interface com a enfermagem, essa amplitude de temas existe nas práticas cotidianas relacionadas ao processo de cuidar e das relações interpessoais e interprofissionais, sendo vista também no empoderamento dos profissionais de enfermagem^(2,18)^ e na defesa dos direitos de pacientes e familiares, trazendo subsídios para o processo de decisões compartilhadas^(26)^. Assim, o princípio da autonomia emerge como um requisito fundamental para dar voz às pessoas cuidadas e à equipe de enfermagem e profissionais de saúde.

Desde os primeiros estudos sobre bioética feminista realizados no Brasil, foi possível observar um protagonismo importante da enfermagem, tanto na produção de trabalhos sobre a temática quanto na aplicação prática de seus conceitos e ideais^(5,6)^. O caráter feminista e crítico dessa corrente teórica da bioética propõe-se a rever o status quo do paternalismo médico e dar voz aos atores historicamente silenciados no contexto clínico e hospitalar^(12,13)^. Consequentemente, evidencia-se na figura da enfermeira a potencialidade transformadora da classe que está intimamente ligada ao cuidado dos indivíduos. Portanto, essa classe seria capaz de absorver, analisar e reprogramar a lógica biomédica com base nas necessidades e nuances reais de cada pessoa e contexto, como defendido por grande parte dos artigos encontrados^(14-18)^.

Como a enfermagem é uma profissão historicamente feminina, marcada pela constante luta por direitos, seu cotidiano é repleto de questões morais e éticas que espelham os debates de gênero e poder presentes no cenário público e político^(13,17)^. Tratando-se de questões inerentes à prática profissional e transformadoras de sua construção e funcionamento, é preciso discutir as relações de trabalho enviesadas pelos papéis de gênero, a desvalorização profissional do cuidado, a presença das interseccionalidades sociais modificadoras do processo de cuidar e de cuidado e o papel de protagonismo da classe na assistência aos processos humanos de reprodução e morte^(13-19)^.

Sendo intrínseco ao trabalho de enfermagem o cuidado contínuo, próximo e centrado no paciente, foi apontada a dominância técnica desses profissionais na organização dos serviços, tanto em aspectos logísticos de distribuição de materiais quanto na ordenação das demandas assistenciais e priorização de urgências^(15,19)^. As enfermeiras foram reconhecidas como as responsáveis pelo cuidado direto e pela manutenção dos elementos fundamentais para a realização plena do trabalho de toda a equipe de saúde^(15,19)^. Porém, contraditoriamente, evidenciou-se que essa classe não tem reconhecida sua responsabilidade moral e sua potencialidade na construção e julgamento dos processos éticos diários, lugar reservado à equipe médica. A análise de tal dinâmica pelas lentes da bioética feminista demonstra a vulnerabilização da classe de enfermagem e o consequente empobrecimento do debate ético na exclusão desse grupo^(12,13,15,18)^.

A sujeição histórica das enfermeiras e seu lugar de submissão e passividade à figura masculina do médico, que detém a moral objetiva, criam a ilusão de que o trabalho de enfermagem é secundário à prática médica^(12,19)^. Nesse sentido, evidenciam-se as relações de poder associadas às questões de gênero no âmbito da assistência à saúde, em que a marginalização do julgamento da enfermagem, ou da ética do cuidado^(19,27)^, significa a dissociação da figura feminina - da enfermeira - do campo de discussão ética^(12,13)^.

A lógica de sujeição da moral feminina originou-se e reproduziu-se amplamente ainda na década de 1970 com os primeiros estudos sobre o desenvolvimento moral e pensamento ético^(27)^. Esse pensamento emergiu em decorrência do fato de que os pesquisadores e os participantes inicialmente incluídos nos estudos pertenciam exclusivamente ao sexo masculino. Construiu-se o entendimento de que os homens seriam capazes de desenvolver-se moralmente assumindo a prerrogativa do processo de tomada de decisões éticas. As mulheres deveriam submeter-se a essa norma, considerando-se que seriam moralmente imaturas. No entanto, em contraposição a essas ideias, mulheres e homens assumiriam posicionamentos distintos com relação à eticidade de suas decisões, sendo que aos homens estaria atribuída a ética da justiça; e às mulheres, a ética do cuidado. O contraste desses dois modelos representa, portanto, a necessidade fundamental de reavaliar os padrões de comportamento e decisão hegemônicos e dar voz a esse grupo que tem sido silenciado. É a partir disso que vem à tona o pluralismo moral tão caro à sociedade contemporânea^(27)^.

Torna-se importante compreender a historicidade na vinculação entre a figura feminina e a figura da enfermeira e a consequente extensão dessa lógica ao ambiente de trabalho em saúde. Tradicionalmente o debate clínico e ético restringia-se aos atores reconhecidos como os detentores da moral, o que os capacitaria para a boa tomada de decisão, observando-se a reprodução de papéis de gênero socialmente estabelecidos nesses cenários. A associação do trabalho de enfermagem a um exercício de bondade e vocação naturais demonstrou sérias consequências na evolução desse sistema: às enfermeiras, destinava-se (e esperava-se delas) a postura de obediência e afeto, porém nunca de decisão e liderança^(28,29)^.

Nota-se a problemática importante nessas relações com a transposição das interseccionalidades de raça, classe e gênero, em que mulheres pretas são ainda mais negligenciadas e silenciadas, sejam elas as usuárias do sistema de saúde, sejam até as enfermeiras da equipe^(17,18)^. Sob a perspectiva interseccional, o construto pode ser definido essencialmente como a investigação da influência das relações de poder estabelecidas pelas questões de gênero, etnia, classe, entre outras na vida social marcada pela diversidade e na vida pessoal cotidiana^(30)^. Sob essa ótica, compreende se que as discriminações de diferentes naturezas e os privilégios não se manifestam isoladamente, mas se constroem e se sobrepõem na leitura integral dos indivíduos.

O racismo institucional sofrido por mulheres pretas e pardas apresenta-se, por exemplo, na dificuldade de acesso de tal população ao sistema de saúde e perpassa todo o processo de cuidado, margeado pelo silêncio das iniquidades^(31)^. Dentro das instituições, onde se espelham as dinâmicas raciais e de gênero, as necessidades de saúde dessas mulheres são ignoradas. Essa situação traz graves consequências, como ilustrado na Pesquisa Nascer no Brasil II: Inquérito Nacional sobre Aborto, Parto e Nascimento^(32)^, na qual se apontou que, entre 2022 e 2023, mais mulheres pretas morreram após o parto em comparação a mulheres brancas, por causas como pré-natal tardio e doenças desenvolvidas durante a gestação, que são fatores preveníveis.

As interseccionalidades de grupos minoritários constroem as relações de poder que ditam os locutores dos discursos^(16)^. Mesmo entre os profissionais de enfermagem, é concreta a marginalização e apagamento histórico e documental de enfermeiras pretas e indígenas que contribuíram imensuravelmente para a profissão. Um exemplo é a enfermeira jamaicana Mary Jane Seacole, contemporânea de Florence Nightingale, que não é lembrada ou citada nas aulas de introdução à enfermagem pelo mundo^(33)^.

Além disso, em eventos recentes como o da pandemia de COVID-19, observaram-se tentativas inúmeras de apagamento e silenciamento do protagonismo feminino, sobretudo de enfermeiras e técnicas de enfermagem pretas e pardas: em publicações jornalísticas e nas redes sociais, a figura masculina e branca foi requisitada e retratada com maior preponderância nos momentos de representação da força de trabalho em saúde^(18)^. Às mulheres, entretanto, é reservado o lugar de menor prestígio social, salários reduzidos e quase nenhum poder de decisão - posição na qual os papéis de “boa profissional” e “boa mulher/esposa/mãe” se confundem^(34)^.

Em cenários de crise sanitária, como o da pandemia de COVID-19^(28)^ e das epidemias de ebola^(35)^, por exemplo, a figura feminina é sacrificada constantemente em nome do cuidado do outro, da comunidade, das crianças e dos homens. No contexto feminino, para além do medo da contaminação e adoecimento, atravessam-se questões relacionadas ao aumento da responsabilidade pelo cuidado do lar e dos filhos, com o fechamento das escolas e creches, requisições de presença e afeto da família, aumento da exposição à violência doméstica, entre outros fatores adoecedores física e psicologicamente. Entre as profissionais de saúde, o cuidado doméstico ampliado ainda é somado ao cuidado profissional da comunidade, que reflete, sobretudo no corpo de enfermagem, as mesmas necessidades e requisições familiares de afeto e escuta. Contraditoriamente, apesar de ser contundente a sobrecarga de trabalho mental e físico dessas profissionais nesse contexto, perpetuam se a falta de reconhecimento e de remuneração adequada e o silenciamento moral da classe^(36)^.

Compreender e reconhecer essa dinâmica permite questionar as razões pelas quais a enfermagem, embora maioria no sistema de saúde e detentora da organização dos processos, permanece lutando pela conquista de espaços e contra as inúmeras tentativas de subordinação profissional^(12,13,15,17,19,20)^. Como aponta Nogueira et al.^(17)^, mesmo após anos de luta feminina coletiva, as enfermeiras encontram dificuldades importantes na tentativa de ocupar lideranças, não somente no campo da saúde como um todo, mas também em seus próprios núcleos de decisão, em que as representações políticas da profissão são ainda dominadas pelo masculino. Mesmo representando a maior parte da força de trabalho em saúde no mundo, cerca de 70%, as mulheres ocupam apenas 25% dos cargos de liderança^(29)^.

Esse quadro, chamado de “anorexia do poder na enfermagem”^(15)^, foi relacionado à assistência que enfraquece a autonomia dos próprios pacientes, os quais, também subordinados às decisões médicas, têm suas vozes silenciadas^(14,20)^. Em contextos como o de parto, aborto e eutanásia^(12-14,20)^, demonstrou-se que essa dinâmica fica clara apesar de serem situações delicadas. Há a subversão da autonomia dos pacientes em contraste com as necessidades e crenças da equipe médica, que dá “a palavra final” na deliberação ética. Em outras palavras, os aspectos sociais e psicológicos que modificam a aplicação do princípio de autonomia do paciente, como bem reconhece as bioeticistas feministas^(12,13)^, são ignorados, de modo que aquelas profissionais capazes de captar tais necessidades no cuidado não participam das decisões.

Nesse quadro, há ainda uma cultura de submissão feminina compulsória, na qual a violência e a dor compõem o processo hospitalar; tal cultura emerge da despolitização da saúde, sendo reafirmada pela prática tecnicista e paternalista biomédica^(14)^. Somente por meio do reconhecimento do papel das questões de gênero, sociais e de classe, ou seja, das interseccionalidades na construção das escolhas e necessidades de cada paciente e da educação em saúde, é possível pensar no pleno exercício da autonomia^(15-17)^. Um dos artigos apontou brilhantemente que não há autonomia sem conhecimento^(13)^.

É fundamental que seja analisada cuidadosamente a aplicação do princípio da autonomia entre as mulheres que se submetem, por exemplo, a determinadas técnicas reprodutivas invasivas, para que a coerção da vontade não se disfarce nos discursos e permita a violência^(13)^. Deve-se analisar não somente a escolha, mas também de que forma a escolha foi feita^(12,13)^. Em contextos nos quais o paternalismo clínico é predominante e os pacientes pouco são informados sobre suas condições de saúde, opções de tratamento e cura disponíveis, as tomadas de decisão são realizadas entre os profissionais, de tal forma que as experiências individuais, sensações e opiniões dos indivíduos são silenciadas e desconsideradas^(37)^.

Compreendeu-se^(14-20)^ que é preciso individualizar os contextos e coletivizar privilégios, sem generalizar os princípios éticos. Alguns artigos demonstraram que as iniciativas devem surgir ainda dentro das faculdades de enfermagem para que aconteçam mudanças concretas no sistema^(14,17)^. Urge promover o debate e a politização atravessando os aspectos de gênero, raça e classe^(17)^, mas sem se restringir aos muros da instituição, pois ainda é necessário que mudanças estruturais e políticas ocorram para permitir a concretude do poder das enfermeiras^(15,18)^.

Além disso, evidenciou-se que o trabalho de politização e conscientização social deve estender-se para a assistência dos grupos em situação de vulnerabilidade, sobretudo por intermédio das enfermeiras enquanto ferramentas por meio das quais os indivíduos no sistema tornem-se agentes de sua própria saúde e detentores de seus direitos^(14-16)^. Um dos artigos^(16)^ relatou uma experiência exitosa nesse sentido, na introdução de elementos culturais da comunidade na assistência prestada pelas enfermeiras, aumentando a adesão do público ao serviço e permitindo a criação de um espaço de aprendizado e conscientização. Para tanto, é necessária a construção de relações honestas no cuidado^(14,16)^.

### Limitações do estudo

Uma das limitações na produção deste artigo foi a delimitação de um período temporal fixo para busca de artigos em bases de dados gratuitas ou de acesso da instituição de ensino. Outra limitação foi a dificuldade de encontrar artigos que tratassem concomitantemente da prática de enfermagem e da bioética feminista. Adicionalmente, a escassez significativa de artigos publicados sobre a temática, mesmo com a aplicação do descritor não controlado “bioética feminista”, restringiu a análise a um número limitado de artigos. Esse fato representa uma lacuna crítica no conhecimento atual, o que dificultou o aprofundamento e discussão dos resultados encontrados.

### Contribuições para a área da Enfermagem

Este estudo busca enriquecer o debate bioético estimulando que seja questionada e repensada a exclusão das vozes de enfermeiras e pacientes - sobretudo aqueles em situação de vulnerabilidade. Espera-se que as ideias discutidas resultem na elaboração e aplicação de iniciativas e em mudanças práticas na assistência para a melhora das condições de trabalho feminina em saúde, bem como na otimização do cuidado com equidade e respeito às diferentes formas de enxergar, vivenciar e compartilhar saúde.

## CONSIDERAÇÕES FINAIS

A enfermagem permanece vítima das questões importantes de gênero, da divisão sexual do trabalho e da marginalização política das mulheres que compõem a classe. A dinâmica ambivalente composta pela vulnerabilização da enfermeira na equipe de saúde e pela notável potencialidade transformadora dessa profissional para com os indivíduos assistidos demonstra a complexidade da discussão. Mais que isso, mostra a importância da politização do grupo na luta por mais espaço e proteção dos interesses e necessidades dos grupos vulnerabilizados.

O reconhecimento das interseccionalidades e o respeito à cultura na assistência permitem a diminuição dos efeitos das desigualdades de gênero, classe e raça. Dessa perspectiva, a valorização do conhecimento coletivo construído pelo diálogo amplo enriquece o debate ético e alcança aqueles que antes se encontravam silenciados. Assim, o fortalecimento da classe de enfermagem também passa pelo fortalecimento da comunidade.

Em suma, a bioética feminista não propõe que as enfermeiras tomem o lugar paternalista ocupado pelo corpo médico e decida pelos indivíduos assistidos. Deve haver um processo equilibrado e equitativo nas deliberações éticas, em que ambos, enfermeiras e pacientes, sejam ouvidos, inseridos em seus diferentes contextos e considerados sujeitos ativos na construção do cuidado.
